# Comparison of image quality and lesion conspicuity between conventional and deep learning reconstruction in gadoxetic acid-enhanced liver MRI

**DOI:** 10.1186/s13244-024-01825-2

**Published:** 2024-10-28

**Authors:** Jeong Hee Yoon, Jeong Eun Lee, So Hyun Park, Jin Young Park, Jae Hyun Kim, Jeong Min Lee

**Affiliations:** 1 Department of Radiology, Seoul National University Hospital and College of Medicine, Seoul, Republic of Korea; 2 Department of Radiology, Chungnam National University Hospital and College of Medicine, Daejeon, Republic of Korea; 3 Department of Radiology, Gachon University Gil Medical Center, Incheon, Republic of Korea; 4 Department of Radiology, Inje University Busan Paik Hospital, Busan, Republic of Korea; 5 Institute of Radiation Medicine, Seoul National University Medical Research Center, Seoul, Republic of Korea

**Keywords:** Liver, Magnetic resonance imaging, Deep learning, Image enhancement

## Abstract

**Objective:**

To compare the image quality and lesion conspicuity of conventional vs deep learning (DL)-based reconstructed three-dimensional T1-weighted images in gadoxetic acid-enhanced liver magnetic resonance imaging (MRI).

**Methods:**

This prospective study (NCT05182099) enrolled participants scheduled for gadoxetic acid-enhanced liver MRI due to suspected focal liver lesions (FLLs) who provided signed informed consent. A liver MRI was conducted using a 3-T scanner. T1-weighted images were reconstructed using both conventional and DL-based (AIR^TM^ Recon DL 3D) reconstruction algorithms. Three radiologists independently reviewed the image quality and lesion conspicuity on a 5-point scale.

**Results:**

Fifty participants (male = 36, mean age 62 ± 11 years) were included for image analysis. The DL-based reconstruction showed significantly higher image quality than conventional images in all phases (3.71–4.40 vs 3.37–3.99, *p* < 0.001 for all), as well as significantly less noise and ringing artifacts than conventional images (*p* < 0.05 for all), while also showing significantly altered image texture (*p* < 0.001 for all). Lesion conspicuity was significantly higher in DL-reconstructed images than in conventional images in the arterial phase (2.15 [95% confidence interval: 1.78, 2.52] vs 2.03 [1.65, 2.40], *p* = 0.036), but no significant difference was observed in the portal venous phase and hepatobiliary phase (*p* > 0.05 for all). There was no significant difference in the figure-of-merit (0.728 in DL vs 0.709 in conventional image, *p* = 0.474).

**Conclusion:**

DL reconstruction provided higher-quality three-dimensional T1-weighted imaging than conventional reconstruction in gadoxetic acid-enhanced liver MRI.

**Critical relevance statement:**

DL reconstruction of 3D T1-weighted images improves image quality and arterial phase lesion conspicuity in gadoxetic acid-enhanced liver MRI compared to conventional reconstruction.

**Key Points:**

- DL reconstruction is feasible for 3D T1-weighted images across different spatial resolutions and phases.
- DL reconstruction showed superior image quality with reduced noise and ringing artifacts.
- Hepatic anatomic structures were more conspicuous on DL-reconstructed images.

**Graphical Abstract:**

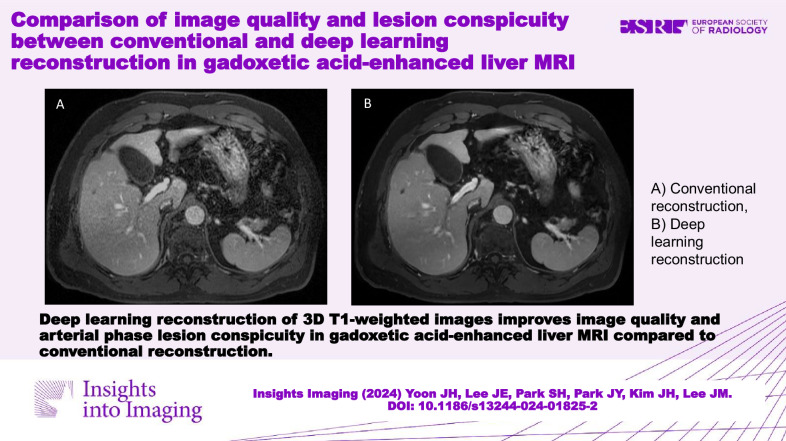

## Introduction

Gadoxetic acid-enhanced liver magnetic resonance imaging (MRI) has been widely used to detect and characterize focal liver lesions (FLLs). Although the superior contrast resolution of gadoxetic acid-enhanced liver MRI offers better diagnostic performance than contrast-enhanced computed tomography, its limitations include limited spatial resolution and sensitivity to artifacts. With the implementation of parallel imaging and a high acceleration factor, spatial resolution can be improved without extending the scan acquisition time [1, 2]. However, applying high acceleration factors to improve higher spatial resolution leads to a decreased signal-to-noise ratio (SNR). It can ultimately reduce image quality and may compromise lesion detectability and diagnostic performance for small FLLs. The combination of parallel imaging and compressed sensing partly resolved this issue, but decreased SNR is still observed [3]. To address this, effective noise reduction strategies that are less computationally demanding and complex compared to iterative reconstruction have become essential. As a result, deep learning (DL)-based image reconstruction is gaining interest as an effective alternative for improving image quality. The DL model learns to transform undersampled *k*-space data into high-quality images by training on large datasets of often fully sampled MRI scans. During reconstruction, the DL model takes the acquired *k*-space data as input and outputs an image with reduced noise and artifacts depending on its training algorithms [4]. DL offers several advantages over conventional parallel imaging and compressed sensing reconstructions. DL can learn more complex image priors that are tailored to specific MRI contrasts and anatomies, enabling better noise suppression and artifact reduction. DL is also computationally efficient, allowing high-quality images to be reconstructed in clinically feasible timeframes. By improving image quality without increasing scan times, DL has the potential to enhance the diagnostic performance of MRI in clinical practice.

Recently, DL-based reconstruction has been shown to have superior denoising capabilities compared to conventional reconstruction methods in liver, brain, and spine imaging [5–7]. However, the application of DL-based reconstruction has been mostly limited to two-dimensional images, including T2-weighted imaging (T2WI) and diffusion-weighted imaging (DWI). Therefore, the current study compared the image quality and lesion conspicuity of three-dimensional (3D) images using conventional or DL reconstruction in patients undergoing gadoxetic acid-enhanced liver MRI.

## Methods

This prospective single-center observational study was approved by the Institutional Review Board of Seoul National University Hospital, and written informed consent was obtained from all participants (NCT05182099). GE Healthcare provided financial support, but the authors maintained full control over the data and information submitted for publication.

### Participants

Between January 2022 and March 2022, we enrolled 54 participants who met the following eligibility criteria: (a) aged 20 years or older; (b) scheduled for gadoxetic acid-enhanced liver MRI to evaluate FLLs, and (c) provided signed informed consent. The exclusion criteria included (a) being under 20 years of age; (b) having an absolute or relative contraindication to gadoxetic acid-enhanced MRI; and (c) having concerns about transient severe motion during the arterial phase due to a previous history (https://classic.clinicaltrials.gov/ct2/show/NCT05182099).

### Image acquisition

All MRI examinations were performed using a 3-T scanner (SIGNA^TM^ Premier, GE Healthcare). Liver MRI comprised heavily T2WI, T2WI, dual-echo images, DWI using two *b*-values (0 s/mm^2^ and 800 s/mm^2^), dynamic imaging (precontrast, arterial, and portal venous phases), and the transitional phase and hepatobiliary phase (HBP) using a fat-suppressed 3D T1W-gradient (GRE) sequence (liver acquisition with volume acquisition, GE Healthcare). Fat suppression was performed with chemical shift selective saturation. The standard dose of gadoxetic acid (0.025 mmol/kg, Primovist or Eovist, Bayer) was administered at a rate of 1 mL/s followed by 20 mL of saline chaser to facilitate the dynamic phases. For the portal venous phase, we captured two sets of images with different spatial resolutions (high resolution and conventional) consecutively, 55 s after contrast agent administration. Three sets of HBP images were acquired at varying spatial resolutions (conventional, high resolution, and small field of view [FOV]) 20 min post-contrast administration. Dual portal venous phase acquisition is for precisely capturing the characteristic features of hepatocellular carcinoma (HCC) including washout and capsule appearance [8]. HBP with different spatial resolutions aims to better depict small FLLs and bile duct anatomy, as well as to improve the diagnostic confidence of readers [1, 9]. Detailed scan parameters are provided in Table 1 and the Supplement.

**Table 1:** Image acquisition of the fat-suppressed T1-weighted gradient sequence

	FOV	TR/TE, (ms)	FA, (°)	Matrix	ST/SP, (mm)	AF^†^
Precontrast	380 × 380	4.0/1.9	11	384 × 300	6/3	2.0
AP^*^	380 × 380	3.1/1.4	11	256 × 240	6/3	3.8
PVP	380 × 380	4.0/1.9	11	384 × 300	6/3	2.0
PVP HR	380 × 380	3.4/1.6	11	300 × 200	2/1	2.2
HBP	380 × 380	4.0/1.9	11	384 × 300	6/3	2.0
HBP HR	380 × 380	4.9/1.7	25	300 × 200	2/1	3.8
HBP small FOV	256 × 256	4.8/1.7	25	256 × 256	3/1.5	3.8

*AF* acceleration factor, *AP* arterial phase, *FA* flip angle, *FOV* field of view, *HBP* hepatobiliary phase, *HR* high resolution, *PVP* portal venous phase, *SP* slice spacing, *ST* slice thickness, *TE* echo time, *TR* repetition time

^*^ Three arterial phases were obtained within a single breath-hold

^†^ ARC 2.0 to 3.8 (2 × 1.0, 2 × 1.1, and 2 × 1.6) was applied and additionally, compressed sensing factor 1.2 was applied to all sequences. The receiver bandwidth was 83.33 kHz in all sequences

### DL-based image reconstruction

The 3D T1-weighted images were also reconstructed using a vendor-provided prototype of a DL algorithm, which is now commercially available (AIR^TM^ Recon DL 3D, GE Healthcare). This algorithm takes *k*-space data as input, and its pipeline incorporates a deep convolutional neural network (CNN) to produce the final image. The CNN comprises approximately 4.4 million trainable parameters distributed across nearly 10,000 kernels [10]. It is designed to work with both Fourier and partial Fourier transformations, including parallel imaging techniques. The algorithm was developed using a supervised learning approach, trained on high spatial resolution, low-noise images, and conventional images. It offers three user-selectable levels of denoising: low, moderate, and high. We chose the high denoising level based on reader preferences during study preparation.

### Image analysis

One fellowship-trained body radiologist (J.M.L.) reviewed the cases and identified participants with severe motion artifacts that could compromise image quality. These cases were excluded in accordance with the study protocol. Subsequently, three fellowship-trained body radiologists (J.E.L., J.Y.P., and S.H.P.) independently evaluated the reconstructed images for the following sequences: T1WI of the precontrast phase, second arterial phase of three arterial phases, portal venous phase, transitional phase, and HBP. Image noise, motion artifacts, ringing artifacts, susceptibility artifacts, and image texture were assessed using a 4-point scale, where a higher score indicated superior image quality with less noise and fewer artifacts [11]. Liver and pancreas edge sharpness, vessel conspicuity, and overall image quality were evaluated using a 5-point scale in a similar manner (Supplement). This broader scale aimed to account for variations not only between reconstruction algorithms but also between standard and high spatial resolutions within each reconstruction method. Reviewers received exemplary images from the literature and clinical cases as references before the evaluation. Additionally, reviewers documented FLL conspicuity and the liver imaging reporting and data system v2018 LR-score [12].

### Reference standard

Two fellowship-trained body radiologists (J.H.Y. and J.H.K.), who did not participate in the review session, evaluated the images alongside 3–6-month follow-up exams and electronic medical records to identify FLLs and their characteristics. The absence of FLLs was confirmed by follow-up CT or MRI conducted within 3–6 months. HCC was diagnosed using follow-up CT/MRI, tumor staining during angiography for transarterial chemoembolization, or an LR-5 classification on contrast-enhanced ultrasound. Metastases were diagnosed through biopsy and positron emission tomography. Benign lesions, such as hemangiomas and cirrhotic nodules, were identified based on their characteristic imaging features and stability across previous and subsequent imaging studies. Further details are provided in the Supplement.

### Statistical analysis

The sample size was calculated to be 45, to attain an 80% power for detecting a difference of 0.56 on average in arterial phase image quality between conventional and DL-reconstructed images. Accounting for an anticipated drop-out rate of 10%, the total number of participants was 50 (Supplement). The paired *t*-test or Wilcoxon rank sum test was used to compare continuous variables between conventional and DL reconstructions. Inter-observer agreement for qualitative analyses was evaluated using Gwet’s AC1 [13, 14]. Weighted jackknife free-response receiver operating characteristic (JAFROC) analyses were performed to evaluate the diagnostic performance of two sequences using the random-reader, random-case method. The lesion detection rates of the reconstruction methods were assessed through generalized estimating equation (GEE) analysis with a binormal distribution and the identity link function. A lesion confidence level of ≥ 3 was considered positive. Lesions scored as LR-4 or LR-5 were defined as a per-lesion HCC diagnosis, and this was analyzed using GEE with a binomial distribution and a logit link function. All statistical analyses were performed using commercially available software (SAS version 9.4; SAS Institute Inc.; MedCalc version 22, MedCalc Software; SPSS version 25.0, SPSS Inc.; irrCAC package in R version 4.3.1, R Foundation for Statistical Computing, 2023; and JAFROC version 4.2.1). A *p*-value of < 0.05 was deemed to indicate statistical significance.

## Results

**Fig. 1:**
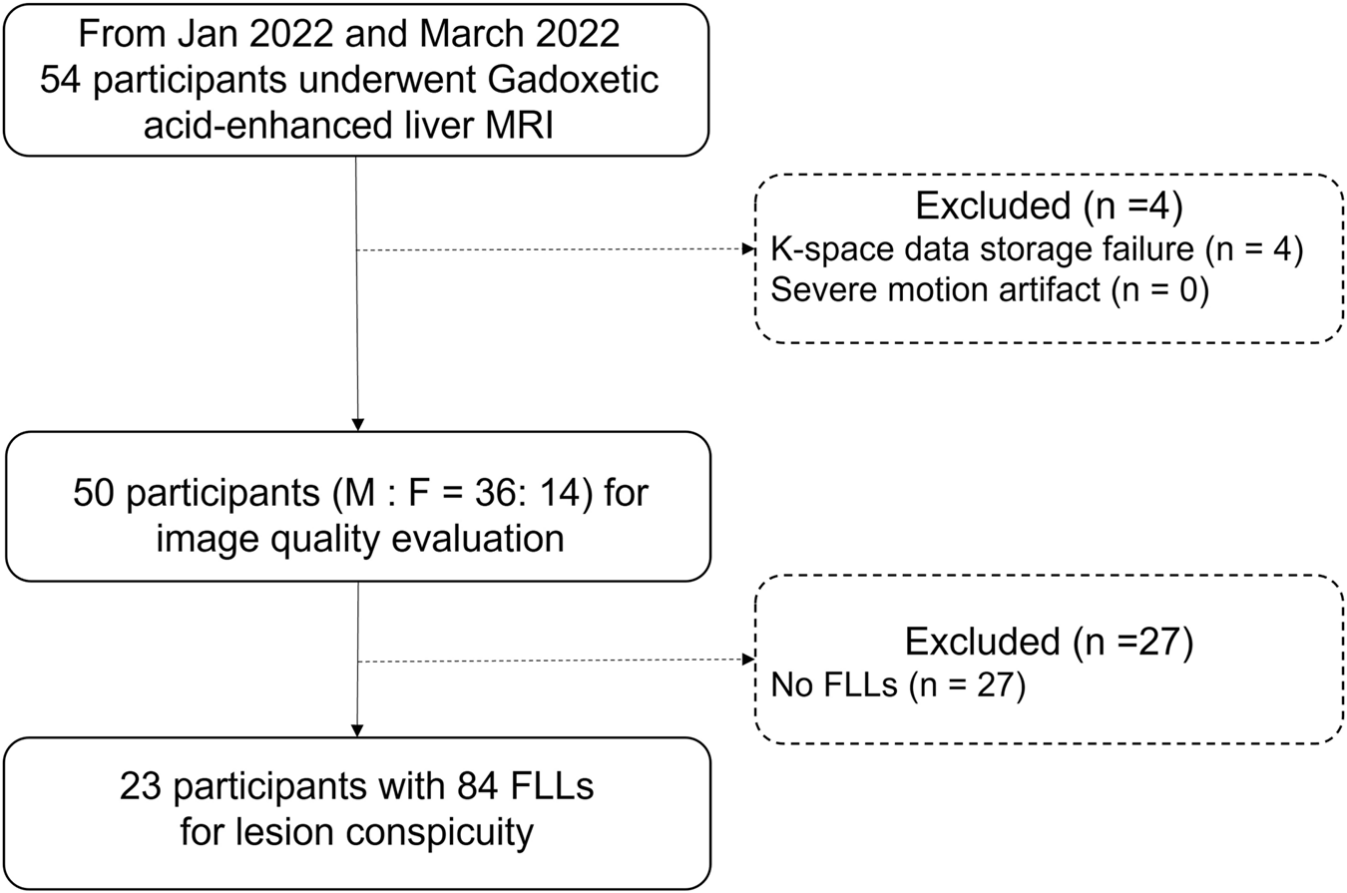
Study flow. FLL, focal liver lesion; MRI, magnetic resonance imaging

Fifty-four participants were initially enrolled in the study, but four were excluded from the analysis due to the loss of *k*-space data necessary for 3D reconstruction (Fig. 1). Consequently, the image analysis included 50 participants (36 males; mean age, 62 ± 11 years). None of the included participants exhibited severe motion artifacts. The mean body mass index was 24.2 ± 2.9 kg/m^2^, and 72% (36/50) of the participants had chronic hepatitis B. Detailed demographic information is provided in Table 2. In total, 84 FLLs were identified in 23 participants. The median size of the lesions was 9.5 mm (range, 3–25 mm). The lesions were categorized as follows: regenerative nodules (*n* = 4), dysplastic nodules (*n* = 26), HCC (*n* = 37), hemangiomas (*n* = 11), one non-specified benign FLL, and metastases (*n* = 5).

**Table 2:** Demographics

Variables	Values
Sex, (male: female)	36:14
Age, (years)	
Female	61 ± 13 years (26, 74)
Male	62 ± 10 years (36, 80)
Body mass index	24.2 ± 2.9 (19.1, 30.9)
Underlying liver disease	
Chronic hepatitis B	72 (36)
Alcoholic liver cirrhosis	8 (4)
Autoimmune hepatitis cirrhosis	6 (3)
Hepatitis C virus cirrhosis	2 (1)
MASH-cirrhosis	2 (1)
Cryptogenic liver cirrhosis	2 (1)
Primary biliary cirrhosis	2 (1)
Liver metastasis*	6 (3)
Albumin, (g/dL)	4.3 ± 0.5 (2.1, 5.0)
Total bilirubin, (mg/dL)	0.7 ± 1.08 (0.5, 4.7)
Prothrombin time, (INR)	1.07 ± 0.1 (0.92, 1.50)
Child-Pugh classification	
Not indicated	6 (3)
Chronic liver disease or class A	90 (45)
Class B	4 (2)

Values are shown as the mean ± standard deviation (range) or percentage (number)

*MASH* Metabolic dysfunction-associated steatohepatitis

* Pancreas/rectal neuroendocrine tumor (*n* = 2) and medullary thyroid cancer (*n* = 1)

### Qualitative image analysis

**Fig. 2:**
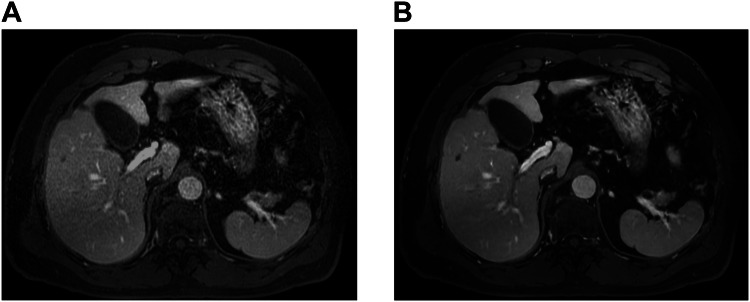
Portal venous phase in a 70-year-old male participant with chronic hepatitis B. Conventional reconstruction of the portal venous phase with a high spatial resolution (**A**) shows substantial image noise, while no or minimal image noise is observed in the liver and peritoneal/subcutaneous fat in a DL-reconstructed image (**B**). The subjective overall image quality was higher in DL reconstruction (**B**) than in conventional reconstruction (**A**)

**Fig. 3:**
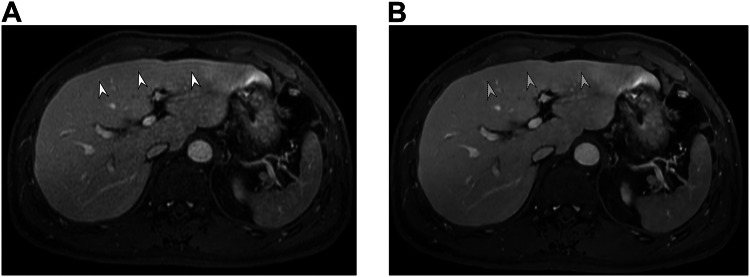
Portal venous phase in a 65-year-old male participant with chronic hepatitis B. A conventional reconstruction image of the portal venous phase with a high spatial resolution (**A**) shows a ringing artifact along the liver edge (arrowheads). In a DL-reconstructed image (**B**), a ringing artifact is not clearly observed (arrowheads with dashed outline)

**Fig. 4:**
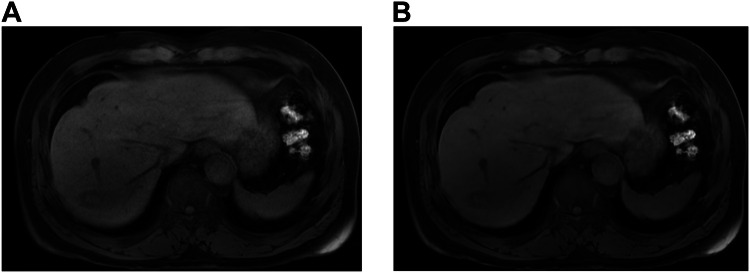
Precontrast phase in a 56-year-old male participant with chronic hepatitis B. Compared with the conventional reconstruction precontrast phase (**A**), a DL-reconstructed image (**B**) shows smoother, altered liver parenchymal texture

The overall image quality was higher in DL-based reconstruction than in conventional images in all phases (3.71–4.40 vs 3.37–3.99, *p* < 0.001 for all) (Table 3). The DL-reconstructed images showed less noise than the conventional images in all phases: 3.87 ± 0.24 vs 3.35 ± 0.28 in the precontrast, 3.59 ± 0.26 vs 3.31 ± 0.27 in the arterial, 3.75 ± 0.23 vs 3.58 ± 0.29 in the portal venous phases, and 3.85 ± 0.28 vs 3.65 ± 0.32 in the HBP (*p* < 0.001 for all). On high-resolution images, the DL reconstruction showed less noise than the conventional images in the portal venous phase (3.82 ± 0.29 vs 3.14 ± 0.30), HBP (3.47 ± 0.46 vs 3.14 ± 0.47), and HBP with small FOV (3.35 ± 0.43 vs 2.89 ± 0.42) (*p* < 0.001 for all) (Fig. 2). Fewer ringing artifacts were observed in the DL-reconstructed images than in the conventional images in the arterial (3.81 ± 0.24 vs 3.67 ± 0.27, *p* = 0.002), portal venous (3.24–3.61 vs 3.73–3.80, *p* < 0.001) phases, and HBP (3.16–3.71 vs 3.53–3.83, *p* < 0.001–0.033) (Fig. 3). The DL-reconstructed images exhibited a smoother image texture than the conventional images in all phases (3.15–3.53 vs 3.76–3.96, *p* < 0.001 for all) (Fig. 4). Susceptibility artifacts showed no differences (*p* = 0.07–0.852), and motion artifacts presented inconsistent differences from phase to phase (Table 3): motion artifact was slightly decreased on DL compared with the conventional images on arterial phase (3.33 ± 0.27 vs 3.16 ± 0.29, *p* < 0.001), but no difference was observed in number of participants with motion artifact score of less than 3 on arterial phase (6% [3/50] in DL and 10% [5/50] in conventional image, *p* = 0.688).

**Table 3:** Intra-individual comparison of image quality between conventional and DL-reconstructed images

	Motion artifacts		Image noise		Ringing artifacts		Susceptibility artifacts		Image texture		Overall image quality	
	Conventional	DL	Conventional	DL	Conventional	DL	Conventional	DL	Conventional	DL	Conventional	DL
Precontrast	2.59 ± 0.33 (1.67, 2.33)	3.13 ± 0.21 (2.67, 3.33)	3.35 ± 0.28 (2.33, 4.0)	3.87 ± 0.24 (3.0, 4.0)	3.64 ± 0.27 (3.0, 4.0)	3.71 ± 0.29 (3.0, 4.0)	3.76 ± 0.29 (2.67, 4.0)	3.83 ± 0.25 (2.67, 4.0)	3.92 ± 0.16 (3.33, 4.0)	3.53 ± 0.33 (3.0, 4.0)	3.73 ± 0.47 (2.0, 5.0)	4.25 ± 0.50 (2.33, 5.0)
* p*-value	< 0.001		< 0.001		0.177		0.070		< 0.001		< 0.001	
AP	3.16 ± 0.29 (2.33, 3.67)	3.33 ± 0.27 (2.67, 4.00)	3.31 ± 0.27 (3.0, 4.0)	3.59 ± 0.26 (3.0, 4.0)	3.67 ± 0.27 (3.0, 4.0)	3.81 ± 0.24 (3.0, 4.0)	3.83 ± 0.23 (3.0, 4.0)	3.87 ± 0.20 (3.33, 4.0)	3.96 ± 0.15 (3.33, 4.0)	3.39 ± 0.23 (3.0, 4.0)	3.47 ± 0.41 (2.33, 4.33)	3.71 ± 0.48 (2.67, 4.67)
* p*-value	< 0.001		< 0.001		0.002		0.280		< 0.001		0.001	
PVP	3.30 ± 0.29 (2.33, 3.67)	3.47 ± 0.23 (2.67, 3.67)	3.58 ± 0.29 (3.0, 4.0)	3.75 ± 0.23 (3.33, 4.0)	3.61 ± 0.30 (3.0, 4.0)	3.80 ± 0.21 (3.33, 4.0)	3.74 ± 0.26 (3.0, 4.0)	3.79 ± 0.22 (3.33, 4.0)	3.76 ± 0.27 (3.0, 4.0)	3.15 ± 0.29 (2.67, 3.67)	3.37 ± 0.34 (2.33, 4.33)	3.71 ± 0.42 (2.67, 4.33)
* p*-value	< 0.001		0.001		< 0.001		0.243		< 0.001		< 0.001	
PVP HR	3.52 ± 0.53 (1.33, 4.0)	3.61 ± 0.43 (2.0, 4.0)	3.14 ± 0.30 (2.0, 3.67)	3.82 ± 0.29 (3.0, 4.33)	3.24 ± 0.27 (3.0, 4.0)	3.73 ± 0.27 (3.0, 4.0)	3.85 ± 0.18 (3.33, 4.0)	3.88 ± 0.21 (3.0, 4.0)	3.79 ± 0.22 (3.0, 4.0)	3.21 ± 0.28 (2.67, 3.67)	3.77 ± 0.60 (1.33, 4.67)	4.40 ± 0.57 (2.67, 5.0)
* p*-value	0.152		< 0.001		< 0.001		0.462		< 0.001		< 0.001	
HBP	3.73 ± 0.39 (2.33, 4.0)	3.91 ± 0.24 (2.67, 4.0)	3.65 ± 0.32 (2.67, 4.0)	3.85 ± 0.28 (3.0, 4.0)	3.71 ± 0.32 (3.0, 4.0)	3.83 ± 0.24 (3.33, 4.0)	3.83 ± 0.26 (3.0, 4.0)	3.84 ± 0.22 (3.33, 4.0)	3.89 ± 0.21 (3.33, 4.0)	3.50 ± 0.29 (2.67, 4.0)	3.99 ± 0.56 (2.0, 4.67)	4.35 ± 0.66 (1.33, 5.0)
* p*-value	0.002		< 0.001		0.014		0.852		< 0.001		< 0.001	
HBP HR	3.77 ± 0.38 (2.33, 4.0)	3.85 ± 0.35 (2.33, 4.0)	3.14 ± 0.47 (2.33, 4.0)	3.47 ± 0.46 (2.33, 4.0)	3.16 ± 0.33 (2.33, 4.0)	3.53 ± 0.36 (3.0, 4.0)	3.86 ± 0.20 (3.33, 4.0)	3.85 ± 0.18 (3.33, 4.0)	3.80 ± 0.24 (3.0, 4.0)	3.34 ± 0.30 (2.67, 4.0)	3.90 ± 0.67 (1.67, 5.0)	4.40 ± 0.79 (1.67, 5.0)
* p*-value	0.146		< 0.001		< 0.001		0.833		< 0.001		< 0.001	
HBP small FOV	3.87 ± 0.24 (3.0, 4.0)	3.93 ± 0.17 (3.33, 4.0)	2.89 ± 0.42 (2.0, 3.67)	3.35 ± 0.43 (2.33, 4.0)	3.68 ± 0.29 (3.0, 4.0)	3.79 ± 0.21 (3.33, 4.0)	3.91 ± 0.15 (3.67, 4.0)	3.88 ± 0.16 (3.67, 4.0)	3.83 ± 0.20 (3.33, 4.0)	3.40 ± 0.30 (2.67, 4.0)	3.85 ± 0.58 (1.67, 5.0)	4.31 ± 0.66 (1.67, 5.0)
* p*-value	0.059		< 0.001		0.033		0.229		< 0.001		< 0.001	

Values are shown as the mean ± standard deviation (range). The *p*-value indicates the difference between the conventional and DL-reconstructed images

*p*-value < 0.05 indicates a statistically significant difference

*AP* arterial phase, *FOV* field of view, *HBP* hepatobiliary phase, *HR* high resolution, *PVP* portal venous phase

### Comparison of anatomical structure and lesion conspicuity between conventional and DL-reconstructed images

**Fig. 5:**
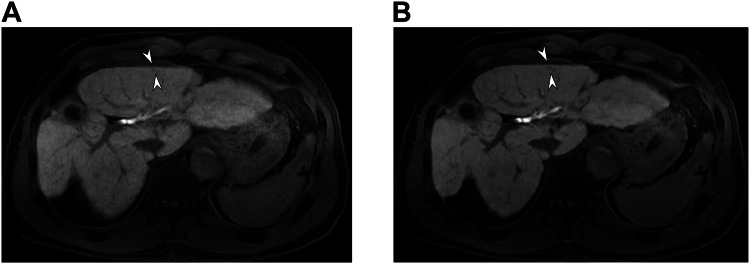
HBP in a 71-year-old male participant with chronic hepatitis B. A HBP image with routine resolution and conventional reconstruction (**A**) barely reveals an approximately 6 mm nodule in liver segment 3, clinically diagnosed as a dysplastic nodule (arrowheads). Compared with the conventional reconstruction (**A**), a DL-reconstructed image (**B**) shows higher lesion conspicuity (arrowheads)

The DL-reconstructed images showed higher conspicuity of the hepatic artery (3.99 ± 0.55 vs 3.65 ± 0.46), portal vein (4.57 ± 0.49 vs 4.03 ± 0.54), and hepatic vein (4.55 ± 0.73 vs 4.28 ± 0.64) than the conventional images (Table 4, *p* < 0.001–0.001). Liver edge sharpness and pancreas contour sharpness also had higher conspicuity in the DL-reconstructed images (Table 4, *p* < 0.001). Lesion conspicuity was higher in the DL-reconstructed images than in the conventional images in the arterial phase (2.15 [95% confidence interval: 1.78–2.52] vs 2.03 [1.65–2.40], *p* = 0.036) (Fig. 5). However, the lesion conspicuity of the DL-reconstructed images was not different from that of the conventional images in the portal venous phase (1.84 vs 1.84, *p* > 0.999) and high-resolution portal venous phase (1.93 vs 1.93, *p* = 0.951). In the HBP, the DL-reconstructed images showed slightly higher lesion conspicuity than the conventional images, but this difference did not reach statistical significance (2.63 vs 2.53, *p* = 0.141 in the HBP, 2.92 vs 2.81, *p* = 0.128 in the high-resolution HBP, and 2.90 vs 2.82, *p* = 0.357 in the HBP with small FOV) (Table 5).

**Table 4:** Intra-individual comparison of organ and vascular conspicuity between conventional and DL-reconstructed images

	Conventional	DL	*p*-value
Precontrast phase			
Pancreas contour sharpness	3.65 ± 0.42 (2.67, 4.33)	4.17 ± 0.56 (2.33, 5.0)	< 0.001
AP			
HA conspicuity	3.65 ± 0.46 (2.67, 4.67)	3.99 ± 0.55 (2.67, 5.0)	< 0.001
PVP			
PV conspicuity	3.26 ± 0.35 (2.67, 4.0)	3.41 ± 0.38 (2.67, 4.33)	0.012
Liver edge sharpness	3.32 ± 0.28 (2.67, 4.0)	3.89 ± 0.32 (3.33, 4.33)	< 0.001
PVP HR			
PV conspicuity	4.03 ± 0.54 (2.33, 5.0)	4.57 ± 0.49 (2.67, 5.0)	< 0.001
Liver edge sharpness	3.90 ± 0.54 (1.33, 5.0)	4.39 ± 0.55 (2.67, 5.0)	< 0.001
HBP			
HV conspicuity	3.81 ± 0.57 (1.67, 4.67)	4.09 ± 0.74 (1.33, 5.0)	0.001
Liver edge sharpness	3.85 ± 0.38 (3.0, 4.33)	4.40 ± 0.36 (3.33, 5.0)	< 0.001
HBP HR			
HV conspicuity	4.28 ± 0.64 (1.67, 5.0)	4.55 ± 0.73 (1.67, 5.0)	0.001
Liver edge sharpness	4.33 ± 0.50 (3.0, 5.0)	4.69 ± 0.53 (2.33, 5.0)	< 0.001
HBP small FOV			
HV conspicuity	4.31 ± 0.70 (1.33, 5.0)	4.57 ± 0.62 (1.67, 5.0)	0.001

Values are shown as the mean ± standard deviation (range). Conspicuity and sharpness were evaluated on a five-point scale. The *p*-value indicates the difference between the conventional and DL-reconstructed images

*p*-value < 0.05 indicates a statistically significant difference

*AP* arterial phase, *FOV* field of view, *HBP* hepatobiliary phase, *HR* high resolution, *HV* hepatic vein, *PV* portal vein, *PVP* portal venous phase

**Table 5:** Intra-individual comparison of lesion conspicuity and lesion detection between conventional and DL-reconstructed images

	Conventional	DL reconstruction	Difference	*p*-value
Lesion conspicuity				
Arterial phase	2.03 [1.65, 2.40]	2.15 [1.78, 2.52]	0.123 [0.008, 0.238]	0.036
PVP	1.84 [1.54, 2.14]	1.84 [1.56, 2.11]	< 0.001 [−0.161, 0.161]	> 0.999
PVP HR	1.93 [1.62, 2.24]	1.93 [1.61, 2.26]	0.004 [−0.123, 0.131]	0.951
HBP	2.53 [2.11, 2.95]	2.63 [2.20, 3.06]	0.103 [−0.034, 0.241]	0.141
HBP HR	2.81 [2.31, 3.31]	2.92 [2.43, 3.40]	0.103 [−0.030, 0.236]	0.128
HBP small FOV	2.82 [2.32, 3.33]	2.90 [2.41, 3.40]	0.079 [−0.089, 0.248]	0.357
Lesion detection				
Figure-of-merit	0.709 [0.630, 0.787]	0.728 [0.667, 0.790]	0.020 [−0.077, 0.116]	0.474
Pooled sensitivity	0.524 [0.378, 0.665]	0.528 [0.375, 0.676]	0.004 [−0.064, 0.071]	0.908
Pooled specificity	0.867 [0.753, 0.933]	0.827 [0.725, 0.896]	−0.040 [−0.113, 0.033]	0.283
Pooled PPV	0.868 [0.771, 0.928]	0.836 [0.743, 0.901]	−0.032 [−0.093, 0.029]	0.303
Pooled NPV	0.520 [0.346, 0.689]	0.510 [0.327, 0.691]	−0.010 [−0.049, 0.030]	0.629

Values are estimates [95% confidence intervals]. *p*-value < 0.05 indicates a statistically significant difference

*PPV* positive predictive value, *NPV* negative predictive value

### Comparison between lesion detectability in DL-reconstructed and conventional images

There was no difference in the figure-of-merit between the two image sets (0.728 [95% CI: 0.667–0.790] in DL-reconstructed vs 0.709 [0.630–0.787] in conventional images, *p* = 0.474) (Table 5). No difference was observed in the sensitivity (57.7% vs 56.8%, *p* = 0.740), specificity (81.4% vs 79.0%, *p* = 0.436), positive predictive value (PPV) (54.2% vs 50.8%, *p* = 0.344), or negative predictive value (NPV) (74.9% vs 72.9%, *p* = 0.320) between the two image sets. Each reviewer’s results are summarized in the Supplement (Table E1).

### Comparison of HCC diagnosis based on DL-reconstructed and conventional images

In a per-lesion analysis, the pooled sensitivity (57.7% in conventional images and 56.8% in DL-reconstructed images) and specificity (81.4% and 79%, respectively) were not different between the DL-reconstructed and conventional images (*p* = 0.740 and 0.436, respectively). The PPVs of the conventional and DL-reconstructed images were 54.2% and 50.8%, respectively, without a statistically significant difference (*p* = 0.344). The NPVs of the conventional and DL-reconstructed images were 83.5% and 82.7%, respectively (*p* = 0.335) (Table E2).

### Inter-observer agreement

The inter-observer agreement was 0.364–0.842 for motion artifacts, 0.485–0.717 for noise, 0.572–0.710 for ringing, 0.626–0.791 for susceptibility, 0.542–0.646 for texture, and 0.276–0.569 for overall image quality. Furthermore, the inter-observer agreement was 0.303 (95% CI: 0.021–0.174) for pancreas edge, 0.380 (95% CI: 0.121–0.289) for hepatic artery conspicuity, 0.290–0.512 for portal vein conspicuity, 0.290–0.700 for liver edge sharpness, and 0.461–0.633 for hepatic vein conspicuity. Details of the inter-observer agreement for each phase are further described in the Supplement and Table E3.

## Discussion

Our study results demonstrated that DL-based reconstruction improved image quality compared to conventional reconstruction in 3D T1-weighted GRE sequences. First, there was a clear reduction in noise with the DL reconstruction, consistent with previous research [5–7]. Additionally, ringing artifacts were substantially reduced in the DL-reconstructed images across multiple phases. The DL-reconstructed images also exhibited greater clarity of anatomical structures than the conventional images, largely due to the reduction in image noise. To date, DL reconstruction has been infrequently applied to 3D imaging, particularly in the upper abdomen [6, 15–17], due to the technical challenges associated with applying DL-based reconstruction algorithms to 3D data such as increased computational requirements, the need for efficient architectures to capture complex spatial relationships, and the scarcity of large, high-quality 3D MRI datasets for training [18]. Furthermore, the greater variability in 3D acquisition parameters, including slice thickness, overlap, and potential inconsistencies between slices, may impede model optimization. These challenges may be addressed through advances in hardware, network design, and data augmentation techniques, as well as by incorporating physics-based domain knowledge and unsupervised learning approaches [4]. Nevertheless, our findings indicate that DL-based reconstruction is feasible for 3D T1WI, which is essential for imaging of the upper abdomen.

The denoising capability of the DL algorithm was more pronounced in high-resolution images. Higher spatial resolution images tend to exhibit increased noise due to smaller pixel sizes and a more granular texture. Notably, the portal venous phase exhibited lower noise scores with routine vs high resolution on conventional reconstruction (3.58 vs 3.14), yet both improved comparably with DL (3.75 vs 3.82). This enhancement was also observed in the HBP with a small FOV, which had an interpolated voxel size of 1 × 1 × 1 mm^3^, where DL reduced the noise (average image noise scores decreased from 2.89 to 3.35). These results support the DL algorithm’s broad applicability across different spatial resolutions and imaging phases. High spatial resolution is often sought to capture fine anatomical details but is accompanied by an increase in image noise, presenting an inherent trade-off. The DL algorithm’s ability to diminish noise while preserving high resolution highlights its potential to deliver detailed images without compromising quality. As a result, DL reconstruction may have the potential to improve the diagnostic performance of body MRI.

The DL-reconstructed images demonstrated better conspicuity of anatomical structures than the conventional images in our study. The liver edge, hepatic artery, portal vein, and hepatic vein were more clearly delineated in the DL-reconstructed images. Furthermore, the contour of the pancreas—a deep-seated, small organ that is often difficult to visualize clearly due to the surrounding bowels—also appeared more defined in the DL-reconstructed precontrast phase, reflecting an additional benefit of DL-reconstructed imaging. It shows the benefit of DL compared with the filtering method, which is traditionally used for denoising. The filtering method is straightforward and effective, yet it inevitably diminishes the effective spatial resolution by attenuating high spatial frequencies that are essential for capturing fine details [19]. Despite the complexity of linear or nonlinear filtering, stochastic methods of noise estimation, and partial differential equation-based methods, achieving effective denoising without image blurring remains challenging, particularly in heterogeneous regions [20]. Therefore, the finding that both image noise and ringing were reduced while preserving lesion conspicuity suggests the potential of DL-based models for clinical application.

We did not observe differences in susceptibility artifacts, most likely due to the distinct nature of the artifacts compared to image noise, which is primarily influenced by the material property, and field strengths and also affected by scan parameters of bandwidths, frequency, and phase encoding directions [19]. Our DL model was not specifically designed to address susceptibility artifacts as they can often be easily recognized and differentiated from pathologies [10]. Future work may address this artifact in addition to noise and ringing artifact reduction. Additionally, there was a slight improvement in motion artifacts, even though they were not explicitly addressed in the CNN model’s training. Our study did not include participants with severe motion artifacts, so the results should not be interpreted as indicative of the DL model’s ability to correct for bulk motion. The observed reduction in motion artifacts may be attributable to the correction of ringing and the sharpening of organ margins, which could lead to a qualitative perception of fewer motion artifacts. However, these benefits were accompanied by changes in image texture, giving the images a plastic or overly smooth appearance, a finding that aligns with previous CT studies comparing conventional and DL reconstructions [21]. The cause of these texture alterations is multifaceted: the DL model we used may smooth out fine details while suppressing image noise, and the specific loss functions employed in the DL model may predispose the algorithm to favor smoother reconstructions. The observed texture alteration highlights the need for careful validation and potential refinement of DL algorithms to ensure they preserve essential diagnostic features while improving image quality. We selected a high level of denoising of three levels (low, medium, and high), and this user-defined level is likely related to the degree of texture alteration. However, we did not observe differences in lesion detection and lesion conspicuity, the probability of losing fine details would be low. Future research should explore the optimal balance between denoising benefits and the users’ preferences.

Although image quality clearly improved and anatomic structures were more sharply delineated with the use of the DL reconstruction, lesion conspicuity was not markedly enhanced, which is inconsistent with a previous study [11, 22]. The reasons for this are not entirely clear, but several hypotheses can be proposed. Firstly, the relatively small number of FLLs in our study may have reduced the statistical power. Secondly, most of our study population faced a high risk for HCC. Consequently, most FLLs (79.8%, 67/84) were either cirrhotic nodules or small HCCs. Cirrhotic nodules are less visible during dynamic phases except for the HBP [23], and small HCCs infrequently exhibit portal washout. This may explain why we did not see a difference in lesion conspicuity during the portal venous phase. Additionally, lesion conspicuity depends on both spatial and contrast resolution. In the HBP, the lesion-to-liver contrast is heightened due to liver parenchymal enhancement. Consequently, we believe that the contrast-to-noise ratio of the lesion would be high regardless of the reconstruction algorithm used. Therefore, the impact of DL reconstruction on lesion conspicuity is likely to be minimal during the HBP. Regarding the diagnosis of HCC, the difference was not statistically significant, which we attribute to the same factors—namely, the absence of portal washout in small HCCs and the adequate contrast-to-noise ratio between liver parenchyma and HCC. We suggest that a larger-scale study should further investigate the potential improvement in diagnostic performance with DL reconstruction.

Our study has several limitations. Firstly, it is a single-center study with a limited number of participants, which may weaken the statistical power for comparing diagnostic performance between the two image sets. However, this was not our primary endpoint, and future studies with a larger population are needed. Secondly, the DL reconstruction level was selected empirically which may be responsible for the texture alteration observed in our study. The optimal level may vary among the organs in the abdomen. Third, the reliability of the DL model should be further tested in variable anatomic organs in patients with variable body habitus and diseases. Furthermore, our study primarily focused on patients at high risk for HCC, which may affect the lesion conspicuity and detection of two data sets. Lastly, the radiologists’ experience and familiarity with DL-reconstructed images might have influenced their assessments. Future studies should involve readers with varying levels of experience as DL reconstruction becomes more popular.

DL reconstruction yielded superior image quality in 3D T1-weighted imaging compared to conventional reconstruction method in gadoxetic acid-enhanced liver MRI.

## Supplementary information

ELECTRONIC SUPPLEMENTARY MATERIAL

## Data Availability

The data used to support the findings of this study are available from the corresponding author upon reasonable request.

## Abbreviations

CNN: Convolutional neural network
DL: Deep learning
FLL: Focal liver lesion
FOV: Field of view
HBP: Hepatobiliary phase
HCC: Hepatocellular carcinoma
MRI: Magnetic resonance imaging
SNR: Signal-to-noise ratio
