# Supplementary material for: Comparison of image quality and lesion conspicuity between conventional and deep learning reconstruction in gadoxetic acid-enhanced liver MRI
Source: Insights Imaging. 2024 Oct 28;15:257. doi: 10.1186/s13244-024-01825-2 (PMC11519238; doi:10.1186/s13244-024-01825-2)
Supplement: Supplementary file 1 — ELECTRONIC SUPPLEMENTARY MATERIAL [file 13244_2024_1825_MOESM1_ESM.pdf]

Comparison of image quality and lesion conspicuity between conventional and deep-learning reconstruction in gadoxetic acid-enhanced liver MRI

ELECTRONIC SUPPLEMENTARY MATERIAL

Image acquisition

Liver magnetic resonance imaging (MRI) includes heavily T2-weighted imaging (T2WI), T2WI, dual echo imaging, and diffusion-weighted imaging (DWI), in addition to dynamic T1-weighted imaging. Heavily T2WI was obtained with a single-shot fast spin echo (SSFSE) sequence without fat saturation using the following scan parameters: field of view (FOV) 380 × 380, repetition time (TR)/echo time (TE) 770/167.7 ms, flip angle (FA) 90°, matrix 352 × 288, number of excitations (NEX) 1.0, and slice thickness (ST)/slice gap (SP) 3/3 mm. Fat-suppressed T2WI was acquired with an FSE sequence at an FOV of 380 × 380, TR/TE 3200/80 ms, matrix 288 × 288, FA 130°, ST/SP 3/3 mm, and an NEX of 1.0. Dual-echo images were captured with the same FOV, TR/TE 4.7/1.4 ms, FA 9°, matrix 352 × 288, and an NEX of 0.7. DWI was performed using three b-values (50, 400, and 800 sec/mm^2) with a NEX of 2.0 in a respiratory triggering manner. T2WI using SSFSE and DWI were reconstructed with a commercially available deep learning (DL) algorithm (AIR™ Recon DL, GE Healthcare) at a console while three-dimensional T1-weighted images were reconstructed with a prototype algorithm as offline, which was not commercially available in South Korea during the study period.

Image analysis

Image noise, motion artifacts, ringing artifacts, susceptibility artifacts, and image texture were evaluated using a 4-point scale. On this scale, a higher score indicated better image quality with less noise and fewer artifacts.

Image noise—A score of 1 indicated non-diagnostic quality due to severe noise that impairs the diagnostic capability of the readers. A score of 2 represented substantial noise that decreased the image quality but did not impair diagnostic performance. A score of 3 was assigned to images with mild quality disturbance due to noise, and a score of 4 denoted images with no or minimal noise.

Motion, ringing and susceptibility artifacts—A score of 1 indicated non-diagnostic (severe artifacts causing impaired diagnostic capability of the readers); a score of 2 denoted substantial artifacts that reduced image quality but did not impair diagnostic performance; a score of 3 corresponded to mild artifacts with a slight decrease in image quality; and a score of 4 reflected the presence of no or minimal artifacts.

Image texture—A score of 1 indicated a non-diagnostic image with severely pixelated texture that impaired the diagnostic capability of the reader; a score of 2 denoted an image with substantial pixelation and an artificial sensation, raising concerns about the loss of normal texture, yet without impairing diagnostic performance; a score of 3 corresponded to an image with mildly pixelated, artificial texture that did not decrease image quality; and a score 4 of represented an image with no or minimal alteration of the texture.

Liver and pancreas edge sharpness, vessel conspicuity, lesion conspicuity, and overall image quality were assessed on a 5-point scale, where a higher score indicated better image quality, increased conspicuity, and sharper contours.

Liver edge sharpness—A score of 1 indicated an extremely blurred edge that hampered

diagnostic performance; a score of 2 corresponded to a moderately blurred edge, resulting in a partially indistinct liver margin; a score of 3 denoted a mildly blurred edge, characterized by a slightly soft liver margin; a score of 4 represented no or minimal blur in the liver margin; and a score of 5 signified no or minimal blurriness with a sharp margin, achieved by high spatial resolution. These assessments were conducted during the portal venous phase and the hepatobiliary phase, both with routine and high spatial resolution imaging.

Pancreas edge sharpness—A score of 1 indicated that the edge was unevaluable due to artifacts, including motion and susceptibility; a score of 2 was assigned when the margin was blurry in 50% or more of the entire pancreas; a score of 3 denoted an identifiable anterior margin of the pancreas with a blurry margin in less than 50% of the organ; a score of 4 signified an identifiable anterior margin of the pancreas, though lacking clear sharpness; and a score of 5 represented a clear anterior margin of the pancreas with its inherent lobulation, distinctly differentiated from the surrounding tissues. This evaluation was conducted during the precontrast phase.

Vessel clarity—A score of 1 indicated that vessels were not delineated due to motion or a low signal-to-noise ratio; a score of 2 signified overall blurred vessel margins; a score of 3 corresponded to average visibility of vessels in the segmental branches of veins and the common and proper hepatic arteries, although the margins of subsegmental branches of arteries and veins were blurred; a score of 4 denoted relatively clear visualization of the peripheral branches of veins and the gastroduodenal artery, as well as the bilateral hepatic arteries; and a score of 5 represented clear visualization of all arteries (from the common hepatic artery to the bilateral intrahepatic arteries) or veins with clear contrast. The hepatic artery was assessed during the arterial phase, while the portal vein was evaluated during the portal venous phase using both routine and high spatial resolutions. The hepatic vein was examined during the hepatobiliary phase with routine and high spatial resolutions, as well as a small field of view (FOV).

Overall image quality—A score of 1 indicated non-diagnostic quality; a score of 2 signified unsatisfactory image quality or substantially low image quality, but without requiring re-examination; a score of 3 represented acceptable image quality, which was average but clinically acceptable; a score of 4 denoted good image quality without significant artifacts; and a score of 5 corresponded to excellent image quality with no or minimal artifacts and high spatial resolution.

Lesion conspicuity—A score of 1 indicated no visualization; a score of 2 signified that the lesion is barely visualized due to low contrast-to-noise ratio between the lesion and the background liver or the presence of substantial noise; a score of 3 denoted partial blurring of the lesion margin with clear contrast, or modest contrast without margin blurriness; a score of 4 represented clear contrast with a distinct border; and a score of 5 corresponded to clear contrast with a distinct border and high spatial resolution.

Reference standard

A total of 84 focal liver lesions (FLLs) were diagnosed in 23 participants (median size 9.5 mm, range 3–25 mm). The lesions were categorized as follows: regenerative nodule (n = 4), dysplastic nodules (n = 26), HCC (n = 37), hemangioma (n = 11), non-specified benign FLL (n = 1), metastasis (n = 5).

HCC—FLLs were diagnosed as HCCs based on imaging criteria: a) Liver Imaging Reporting and Data System (LI-RADS) scores 4-5 (LR-4 or -5) [1] on computed tomography (CT) or MRI with tumor staining on cone-beam CT for transarterial chemoembolization followed by lipiodol uptake [2]; b) LR-4 or -5 nodule with US visibility on ultrasonography; c) LR-4 or -5 nodule showing tumor progression or regression after chemotherapy or recurrence after locoregional therapy; d) or LR-5 on contrast-enhanced ultrasonography [3]. We also considered FLLs with LR-M features as HCC in patients with a history of HCC and tumor marker elevation (alpha-

fetoprotein and/or protein induced by vitamin K absence).

Cirrhotic nodules— Dysplastic nodules are defined as lesions that exhibit defects in the HBP without arterial phase hyperenhancement (APHE) and remain stable for a period exceeding 1 year [4]. Regenerative nodules were defined as nodules that showed hyperintensity in the HBP that remained stable for more than 1 year.

Non-specific benign FLL— One FLL showed strong APHE and an HBP defect, and remained stable for more than 2 years. The clinical diagnosis was bile duct adenoma, but it was not histologically confirmed. We considered this FLL to be a benign, non-specific lesion.

Hemangiomas—Hemangiomas were clinically diagnosed based on their characteristic features, including bright intensity on T2-weighted imaging and a peripheral nodular enhancement pattern on contrast-enhanced CT or dynamic MRI, as well as no significant interval change during follow-up [5].

Metastasis— All metastases were diagnosed with biopsy.

Sample size calculation

One radiologist (J.M.L.) evaluated the image quality of arterial phase images with conventional and DL reconstruction in 20 patients who underwent gadoxetic acid-enhanced liver MRI using a 3T scanner between May 2021 and June 2021. The image quality was graded on a five-point scale, with higher scores indicating better image quality. The mean values were 4.0 ± 0.77 for DL-reconstructed images and 3.44 ± 1.06 for conventional images. The Type I and II errors were set at 0.05 and 0.2, respectively, and the calculated sample size was 45. Assuming a dropout rate of 10%, which includes cases of newly detected claustrophobia or newly developed transient severe motion artifacts, a total of 50 participants are needed.

Inter-observer agreement

Inter-observer agreement (Gwet's AC1) for pancreas edge sharpness on the precontrast phase was 0.303 (95% confidence interval [CI]: 0.021–0.174), and 0.380 (95% CI: 0.121–0.289) on the arterial phase. For the portal venous phase with high resolution, inter-observer agreement was 0.512 (95% CI: 0.344–0.506) for portal vein conspicuity and 0.549 (95% CI: 0.391–0.554) for liver edge sharpness. On the routine portal venous phase, the agreement was 0.290 (95% CI: 0.027–0.139) for portal vein conspicuity and 0.290 (95% CI: 0.022–0.159) for liver edge sharpness. Regarding hepatic vein conspicuity, inter-observer agreement was 0.461 (95% CI: 0.279–0.44) on the hepatobiliary phase (HBP) with routine resolution, 0.633 (95% CI: 0.488–0.656) on HBP with high resolution, and 0.558 (95% CI: 0.424–0.606) on HBP with a small FOV. For liver edge sharpness, the agreement was 0.394 (95% CI: 0.174–0.311) on HBP with routine resolution and 0.700 (95% CI: 0.55–0.72) on HBP with high resolution. Inter-observer agreement for motion artifact, image noise, susceptibility artifact, ringing artifact, image texture, and overall image quality on each phase is summarized in Table E3.

REFERENCES

1. Chernyak V, Fowler KJ, Do RKG, et al (2023) LI-RADS: Looking Back, Looking Forward. Radiology 222801. https://doi.org/10.1148/radiol.222801

2. Yu MH, Kim JH, Yoon J-H, et al (2014) Small (≤1-cm) Hepatocellular Carcinoma: Diagnostic Performance and Imaging Features at Gadoxetic Acid–enhanced MR Imaging. Radiology 271:748–760. https://doi.org/10.1148/radiol.14131996

3. Wilson SR, Lyshchik A, Piscaglia F, et al (2018) CEUS LI-RADS: algorithm, implementation, and key differences from CT/MRI. Abdom Radiol 43:127–142. https://doi.org/10.1007/s00261-017-1250-0

4. Choi JW, Lee JM, Kim SJ, et al (2013) Hepatocellular Carcinoma: Imaging Patterns on Gadoxetic Acid–enhanced MR Images and Their Value as an Imaging Biomarker. Radiology 267:776–786. https://doi.org/10.1148/radiol.13120775

5. Anderson SW, Kruskal JB, Kane RA (2009) Benign Hepatic Tumors and Iatrogenic Pseudotumors. RadioGraphics 29:211–229. https://doi.org/10.1148/rg.291085099

Table E1. Comparison between lesion detection on conventional and deep learning (DL)-reconstructed images in each reader

	Conventional	Deep learning	Diff [95% CI]	*P*-value
	Estimate [95% CI]	Estimate [95% CI]	DL – Conventional	
**Sensitivity**				
Reader 1	0.500 [0.346, 0.654]	0.500 [0.368, 0.632]	<0.001 [-0.087, 0.087]	
Reader 2	0.583 [0.411, 0.738]	0.595 [0.432, 0.740]	0.012 [-0.092, 0.116]	
Reader 3	0.488 [0.326, 0.653]	0.488 [0.286, 0.694]	<0.001 [-0.165, 0.165]	
**Specificity**				
Reader 1	0.960 [0.879, 0.988]	0.940 [0.853, 0.977]	-0.020 [-0.059, 0.019]	
Reader 2	0.740 [0.530, 0.878]	0.620 [0.427, 0.781]	-0.120 [-0.324, 0.084]	
Reader 3	0.900 [0.779, 0.958]	0.920 [0.796, 0.971]	0.020 [-0.084, 0.124]	
**PPV**				
Reader 1	0.955 [0.871, 0.985]	0.933 [0.837, 0.974]	-0.021 [-0.065, 0.023]	
Reader 2	0.790 [0.641, 0.888]	0.725 [0.592, 0.826]	-0.066 [-0.181, 0.049]	
Reader 3	0.891 [0.776, 0.951]	0.911 [0.778, 0.968]	0.020 [-0.091, 0.131]	
**NPV**				
Reader 1	0.533 [0.347, 0.711]	0.528 [0.358, 0.691]	-0.005 [-0.049, 0.039]	
Reader 2	0.514 [0.331, 0.693]	0.477 [0.291, 0.670]	-0.037 [-0.127, 0.053]	
Reader 3	0.511 [0.343, 0.677]	0.517 [0.311, 0.717]	0.005 [-0.084, 0.095]	

Note—. Values are estimated [95% confidence intervals]. PPV = positive predictive value, NPV = negative predictive value

Table E2. Comparison between hepatocellular carcinoma diagnosis on a per-lesion basis using conventional and deep learning (DL)-reconstructed images

	Conventional	DL	Diff [95% CI]	*P*-value
	Estimate [95% CI]	Estimate [95% CI]	DL - Conventional	
**Sensitivity**				
Pooled	0.577 [0.377, 0.754]	0.568 [0.356, 0.757]	-0.009 [-0.062, 0.044]	0.740
Reader 1	0.486 [0.282, 0.696]	0.514 [0.317, 0.706]	0.027 [-0.017, 0.071]	
Reader 2	0.622 [0.365, 0.824]	0.622 [0.361, 0.827]	<0.001 [-0.106, 0.106]	
Reader 3	0.622 [0.434, 0.778]	0.568 [0.301, 0.800]	-0.054 [-0.199, 0.091]	
**Specificity**				
Pooled	0.814 [0.706, 0.889]	0.790 [0.707, 0.855]	-0.024 [-0.085, 0.037]	0.436
Reader 1	0.907 [0.826, 0.953]	0.918 [0.852, 0.955]	0.010 [-0.008, 0.029]	
Reader 2	0.660 [0.482, 0.802]	0.588 [0.458, 0.706]	-0.072 [-0.222, 0.077]	
Reader 3	0.876 [0.770, 0.938]	0.866 [0.763, 0.929]	-0.010 [-0.071, 0.051]	
**PPV**				
Pooled	0.542 [0.341, 0.731]	0.508 [0.320, 0.693]	-0.034 [-0.105, 0.037]	0.344
Reader 1	0.667 [0.433, 0.840]	0.704 [0.504, 0.848]	0.037 [-0.015, 0.089]	
Reader 2	0.411 [0.228, 0.622]	0.365 [0.212, 0.552]	-0.046 [-0.146, 0.054]	
Reader 3	0.657 [0.424, 0.833]	0.618 [0.353, 0.827]	-0.039 [-0.144, 0.065]	
**NPV**				
Pooled	0.835 [0.699, 0.916]	0.827 [0.696, 0.909]	-0.007 [-0.022, 0.007]	0.335
Reader 1	0.822 [0.685, 0.908]	0.832 [0.702, 0.912]	0.009 [-0.004, 0.023]	
Reader 2	0.821 [0.639, 0.922]	0.803 [0.636, 0.905]	-0.018 [-0.065, 0.030]	

Reader 3	0.859 [0.739, 0.929]	0.840 [0.700, 0.922]	-0.019 [-0.059, 0.022]	
**Accuracy**				
Pooled	0.749 [0.646, 0.830]	0.729 [0.651, 0.795]	-0.020 [-0.059, 0.019]	0.320
Reader 1	0.791 [0.696, 0.862]	0.806 [0.718, 0.871]	0.015 [-0.002, 0.032]	
Reader 2	0.649 [0.496, 0.777]	0.597 [0.498, 0.689]	-0.052 [-0.159, 0.054]	
Reader 3	0.806 [0.703, 0.879]	0.784 [0.677, 0.862]	-0.022 [-0.066, 0.022]	

Note—. Values are estimates [95% confidence intervals]. PPV = positive predictive value, NPV = negative predictive value

Table E3. Interobserver agreement

	Motion artifact	Image noise	Ringing artifact	Susceptibility artifact	Image texture	Overall image quality
**Precontrast**	0.572 (0.33, 0.515)	0.620 (0.402, 0.596)	0.572 (0.36, 0.548)	0.70 (0.58, 0.746)	0.646 (0.459, 0.668)	0.313 (0.122, 0.239)
**AP**	0.364 (0.038, 0.213)	0.485 (0.222, 0.387)	0.623 (0.235, 0.528)	0.758 (0.567, 0.784)	0.646 (0.444, 0.555)	0.364 (0.175, 0.318)
**PVP**	0.367 (0.067, 0.195)	0.556 (0.045, 0.355)	0.60 (0.158, 0.456)	0.626 (0.451, 0.639)	0.481 (0.266, 0.457)	0.276 (0.009–0.126)
**PVP HR**	0.586 (0.416, 0.588)	0.637 (0.494, 0.665)	0.633 (0.417, 0.60)	0.764 (0.59, 0.796)	0.549 (0.288, 0.485)	0.485 (0.305, 0.467)
**HBP**	0.795 (0.68, 0.843)	0.717 (0.564, 0.743)	0.710 (0.415, 0.685)	0.754 (0.633, 0.801)	0.593 (0.387, 0.588)	0.380 (0.203, 0.325)
**HBP HR**	0.788 (0.668, 0.84)	0.572 (0.298, 0.502)	0.677 (0.483, 0.666)	0.737 (0.54, 0.762)	0.545 (0.298, 0.49)	0.570 (0.41, 0.567)
**HBP Small**	0.842 (0.756,	0.519 (0.232,	0.603 (0.196,	0.791 (0.652,	0.542 (0.301,	0.542 (0.374,

**FOV**	0.896)	0.413)	0.493)	0.835)	0.495)	0.538)

Note—. Values in parentheses are 95% confidence intervals. AP = arterial phase, FOV = field of view, HBP = hepatobiliary phase, HR = high resolution, PVP = portal venous phase.
